# Synchronous colon cancer presenting as toxic megacolon in a patient with ulcerative colitis: A case report

**DOI:** 10.1016/j.ijscr.2023.108984

**Published:** 2023-10-24

**Authors:** Emmanuel Luciano, Sarah Macek, Felipe Pacheco, Wael Solh

**Affiliations:** Department of Surgery, Central Michigan University College of Medicine, United States of America

**Keywords:** Case report, colon cancer, Ulcerative colitis, Toxic megacolon

## Abstract

**Introduction and importance:**

The incidence of colorectal cancer in patients with inflammatory bowel disease is greater than the general population. Of those with inflammatory bowel disease, synchronous cancers are more common in ulcerative colitis than in Crohn's disease. It is rare for synchronous cancer to present as toxic megacolon in a patient with concomitant inflammatory bowel disease, specifically ulcerative colitis.

**Case presentation:**

In this report, we describe the clinical presentation of a 22-year-old female, who presented with toxic megacolon ultimately requiring total abdominal colectomy with end-ileostomy and a final pathology of two synchronous colon cancers, despite normal colonoscopy one year prior. The postoperative period was unremarkable, and the patient was referred to medical oncology to pursue adjuvant treatment.

**Clinical discussion:**

Due to the increased incidence of colorectal cancer in patients with ulcerative colitis, screening colonoscopies are typically recommended at more frequent intervals than the general population. Toxic megacolon as the presentation for colon cancer in patients with underlying ulcerative colitis is exceedingly rare. To our knowledge, this is the first case reported of synchronous colon cancer presenting as toxic megacolon in a patient with ulcerative colitis and recent negative screening colonoscopy.

**Conclusion:**

Colorectal cancer should always be high in the differential diagnosis for patients with ulcerative colitis regardless of the age. The principles of oncologic resection for colorectal cancer should be followed during colonic resections in patients with ulcerative colitis, even in the acute setting.

## Highlights

• Colon cancer presenting as toxic megacolon.
• Synchronous colon cancer in a patient with ulcerative colitis.
• Synchronous colon cancer one year after a normal screening colonoscopy.

## 1 Introduction and importance

There is a well-established association of colorectal cancer (CRC) with inflammatory bowel disease, particularly in ulcerative colitis (UC) [1]. The extent of the risk for CRC in patients with UC varies considerably in the current literature [1]. However, the most important risk factors in UC-associated CRC include duration and extent of disease [1]. Furthermore, synchronous colorectal carcinoma has been reported in patients with UC [2]. Of these synchronous cancers, the most common sites of occurrence include the proximal colon such as ascending and transverse [2]. With respect to screening for CRC, most societies recommend surveillance ranging from 1 to 5 years based on risk factors including history of dysplasia, family history, active inflammation, and anatomic pathologies such as inflammatory pseudopolyps, or strictures [3]. However, all societies recommend ongoing surveillance colonoscopies for patients with inflammatory bowel disease such as UC or Crohn's disease with involvement of one-third or more of the colon [3]. Additionally, toxic megacolon, which is defined as severe colonic dilation associated with colitis, is associated with inflammatory bowel disease such as UC and Crohn's [5]. However, while colon cancer is a possible etiology of obstruction resulting in toxic megacolon, it is rare and not usually associated with toxic megacolon. We present a case report of a 22-year-old female, with known UC who presented with toxic megacolon in the setting of two synchronous colon cancers in the ascending and transverse colon despite a normal screening colonoscopy one year prior. This work has been reported in line with the SCARE 2020 criteria [4].

## 2 Case presentation

The patient is a 22-year-old female who was diagnosed with UC at the age of 5. This patient had been intermittently compliant with treatments including steroids and biologic agents. One year prior to presentation, she underwent a screening colonoscopy with biopsies of the terminal ileum, colon, and rectum. Gross colonoscopy demonstrated a normal-appearing terminal ileum, colon, distal rectum, and anal verge. Pathology demonstrated colonic mucosa with changes suggestive of quiescent inflammatory bowel disease and no dysplasia.

**Image 1:**
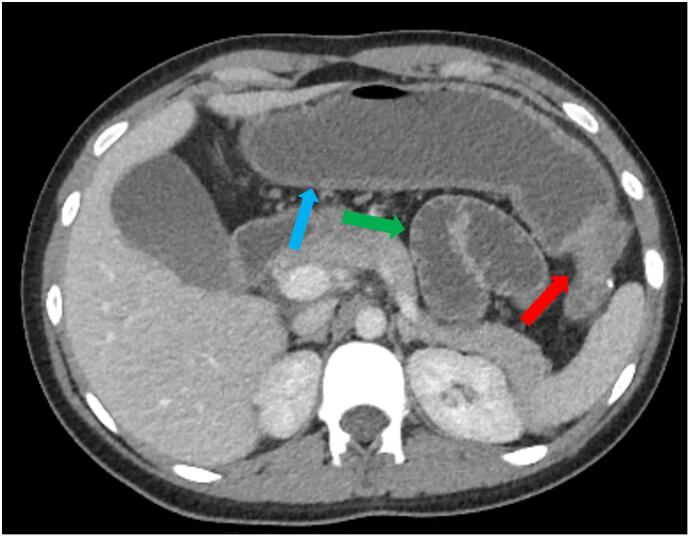
Axial CT of the abdomen and pelvis showing distended transverse colon with wall thickening (blue arrow), focal narrowing at the splenic flexure (red arrow) with distended small bowel (green arrow).

**Image 2:**
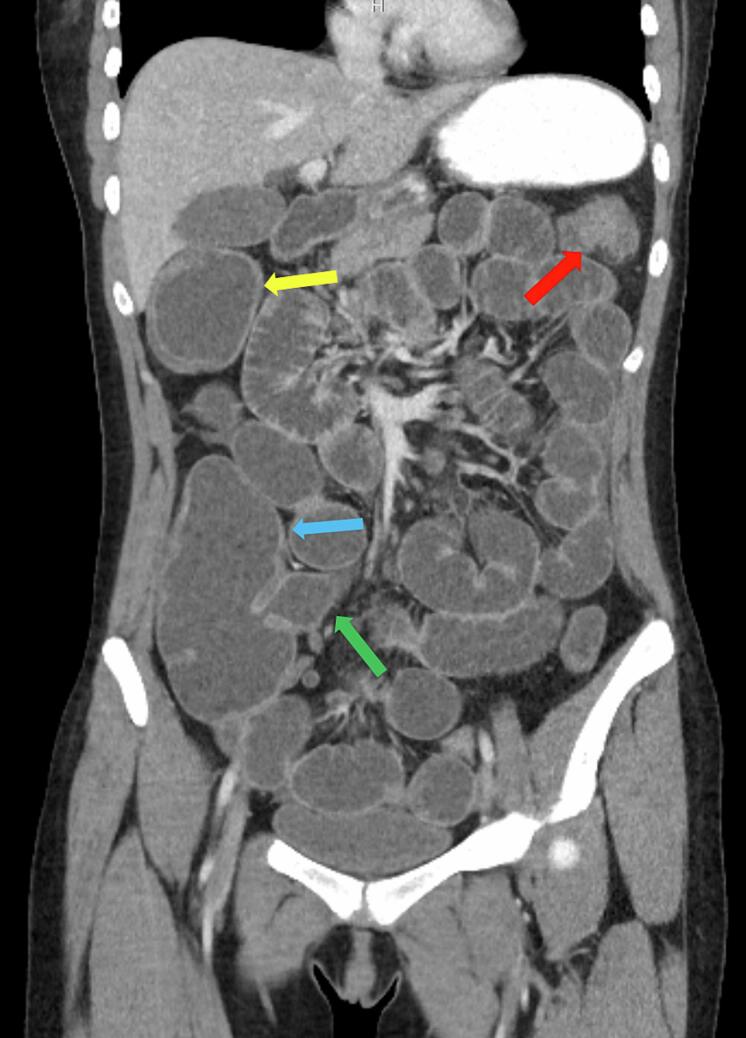
Coronal CT of the abdomen and pelvis showing distended hepatic flexure with wall thickening (yellow arrow), distended ascending colon with wall thickening (blue arrow), focal narrowing at the splenic flexure (red arrow) with distended terminal ileum (green arrow).

**Image 3:**
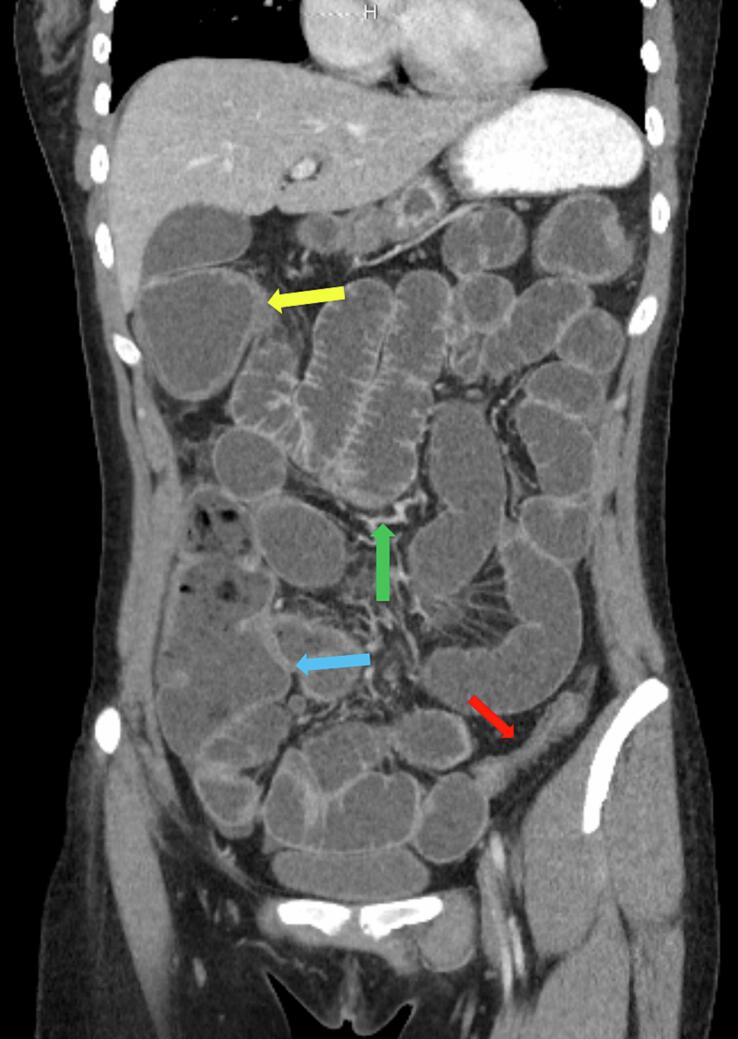
Coronal CT of the abdomen and pelvis showing distended hepatic flexure with wall thickening (yellow arrow), distended ascending colon with wall thickening (blue arrow), focal narrowing and thickening of the sigmoid colon (red arrow) with distended small bowel (green arrow).

The patient presented to the emergency department with complaints of abdominal pain, nausea, and bilious emesis. On admission, she was tachycardic, mildly hypertensive and afebrile. Physical exam was notable for diffuse abdominal tenderness and mild distention without guarding or peritoneal signs. She had mild leukocytosis and lactic acidemia. A computed tomography (CT) of the abdomen and pelvis was performed which demonstrated acute colonic obstruction with focal narrowing at the splenic flexure suggestive of stricture (see Image 1, Image 2) and thickening of the sigmoid colon (see Image 3).

Due to the concerning clinical presentation and imaging findings the decision to take the patient to the operating room for emergent exploration was made. A standard midline laparotomy incision was performed. During exploration of the peritoneal cavity, classic findings of toxic megacolon were identified. The right and transverse colon were distended with patchy areas of transmural necrosis. A mass involving the distal transverse colon was palpated. The rectosigmoid area was markedly indurated and thickened. Due to the previously mentioned findings and the underlying history of UC, we elected to perform a total abdominal colectomy with end-ileostomy. There were no postoperative complications, and the patient was discharged on postoperative day 4. The procedure was performed at a teaching hospital by the senior colorectal surgery attending.

The pathology report demonstrated synchronous adenocarcinoma involving the ascending colon and transverse colon; 3 cm tumor in the ascending colon grade 1 with extension into pericolonic tissue and 4 cm tumor in transverse colon grade 1. Final staging was determined to be stage II synchronous colon cancer pT3N0. Genetic testing was performed and was negative for Lynch syndrome.

The patient was referred to medical oncology and the decision was made to proceed with adjuvant chemotherapy with Oxaliplatin, 5-fluorouracil and folinic acid.

## 3 Clinical discussion

Colorectal cancer in patients with ulcerative colitis is not uncommon [1]. Additionally, among the inflammatory bowel diseases, synchronous cancers are more common in UC, particularly in the proximal colon [2]. Furthermore, in these patients, screening colonoscopies are typically recommended at more frequent intervals than the general population to evaluate for disease progression and development of CRC [3]. Toxic megacolon is another acute disease process that is not uncommon in patients with inflammatory bowel disease, such as UC [5].

In this case, a 22-year-old female with a 17-year history of UC developed two synchronous colon cancers in the ascending and transverse colon one year after a screening colonoscopy which demonstrated a grossly appearing normal colon and biopsies that demonstrated quiescent inflammatory bowel disease without dysplasia. Thus, despite receiving a colonoscopy a year prior, which did not demonstrate any abnormality, she developed two synchronous colon cancers which ultimately presented as toxic megacolon, prompting total abdominal colectomy. Her CT demonstrated marked distention of the ascending and transverse colon, and small bowel with associated narrowing and thickening of the sigmoid. Her resultant cancerous lesions were found in the ascending and transverse colon, of which the distention was significant. To our knowledge, this is the first case reported of synchronous colon cancer presenting as toxic megacolon in a patient with ulcerative colitis and recent negative screening colonoscopy.

One case study in particular by Dumitru et al., discuss three cases of toxic megacolon, one of which occurred in a patient with colon cancer. However, in these patients, the culprit of the toxic megacolon was not inflammatory bowel disease, but rather the development of *clostridium difficile* colitis [5].

Another case study by Rodrigo et al. present the case of a 74-year-old male with toxic megacolon and findings of a tumor in the rectosigmoid junction and underwent left hemicolectomy [6]. In this case, an additional synchronous tumor was found and resected during the creation of the ostomy [6]. This patient also presented with toxic megacolon and was found to have a synchronous colon cancer; however, this patient was of older age and did not have any history of inflammatory bowel disease. In this case, our patient was young, 22-year-old, and had UC in addition to two synchronous colon cancers found in postoperative pathology despite a normal screening colonoscopy one year prior. However, it is worth mentioning that no genomic studies of random mucosal sampling were undertaken which may have identified underlying dysplasia.

It is rare in our case for this young 22-year-old female to have developed two synchronous colon cancers one year after normal screening colonoscopy. However, of much greater significance is her presentation of toxic megacolon, as a result of obstruction from her colon cancer.

## 4 Conclusion

In summary, this is a unique case of an incidentally found synchronous colon cancer in a young patient presenting with a toxic megacolon despite a normal screening colonoscopy one year prior to presentation. Fortunately, this patient's diagnosis of two synchronous colon cancers with total abdominal colectomy and end-ileostomy did not result in any lymph node involvement and she was subsequently treated with adjuvant chemotherapy successfully. It is very important to mention that the principles of oncologic resection for colorectal cancer should be followed during colonic resections in patients with ulcerative colitis, even in the acute setting.

## Consent

Written informed consent was obtained from the patient for publication of this case report and accompanying images. A copy of the written consent is available for review by the Editor-in-Chief of this journal on request.

## Provenance and peer review

Not commissioned, externally peer-reviewed.

## Ethical approval

None required. This was an isolated case report done with the consent of the patient. No further research studies are being pursued.

## Funding

No external funding was available for this study.

## Author contribution

Emmanuel Luciano, MD; conceptualization, methodology, writing original draft and final review and editing.

Sarah Macek; conceptualization, methodology, writing original draft and final review and editing.

Felipe Pacheco, MD; final review and editing.

Wael Solh, MD; final review and editing.

## Guarantor

Emmanuel Luciano, MD.

## Research registration number

N/a.

## Conflict of interest statement

None declared.
